# Increased monocytic myeloid-derived suppressor cells in type 2 diabetes correlate with hyperglycemic and was a risk factor of infection and tumor occurrence

**DOI:** 10.1038/s41598-024-54496-w

**Published:** 2024-02-22

**Authors:** Ji Zhou, Mengjie Zhang, Xiaodi Ju, Huiping Wang, Hao Xiao, Zhimin Zhai, Xing Zhong, Jingfang Hong

**Affiliations:** 1 Department of Epidemiology and Health Statistics, School of Public Health, Anhui Medical University, Hefei, 230032 Anhui China; 2 Department of Endocrinology, The Second Affiliated Hospital of Anhui Medical University, 678 Furong Road, Hefei, 230601 Anhui China; 3 Hematologic Department/Hematologic Disease Research Center, the Second Affiliated Hospital of Anhui Medical University, Hefei, 230601 Anhui China; 4 School of Nursing, Anhui Medical University, Hefei, 230032 Anhui China; 5 Nursing International Collaboration Research Center of Anhui Province, Hefei, 230601 Anhui China; 6 Department of Endocrinology, Fuyang People’s Hospital, Fuyang, 236000 Anhui China

**Keywords:** Immunology, Endocrinology

## Abstract

To investigate the frequency of monocytic myeloid-derived suppressor cells (M-MDSCs) in type 2 diabetes mellitus (T2DM) patients and explore the potential associations between M-MDSCs, glycemic control, and the occurrence of infections and tumor. 102 healthy and 77 T2DM individuals were enrolled. We assessed the M-MDSCs frequency, levels of fasting plasma glucose (FPG), haemoglobin A1c (HbA1c), and other relevant indicators. Each patient underwent a follow-up of at least 6 months after M-MDSCs detection. The M-MDSCs frequency was significantly higher in patients with poor glycemic control (PGC) compared to the healthy population (*P* < 0.001), whereas there was no significant difference between patients with good glycemic control and the healthy (*P* > 0.05). There was a positive correlation between the M-MDSCs frequency and FPG, HbA1c (*R* = 0.517 and 0.315, *P* < 0.001, respectively). T2DM patients with abnormally increased M-MDSCs have a higher incidence of infection and tumor (48.57% and 11.43% respectively). Our results shed new light on the pathogenesis of T2DM, help to understand why T2DM patients are susceptible to infection and tumor and providing novel insights for future prevention and treatment of T2DM.

## Introduction

Type 2 diabetes mellitus (T2DM) is a chronic metabolic disorder characterized by persistent hyperglycemia and closely linked to the epidemic of obesity. The main cause of T2DM is insulin resistance, initially pancreatic β-cells maintain glucose homeostasis by an increase in insulin production, but over time, insulin secretion decreases and even pancreatic β-cell failure, resulting in varying degrees of hyperglycemia^1–5^. According to the International Diabetes Federation (IDF 2021) etc., approximately 537 million adults (20–79 years) suffer from diabetes and 6.7 million patients died from diabetes in 2021. T2DM is the most common type of diabetes, accounting for approximately 90% of all diabetes cases. Epidemiological data and various studies indicate that, in addition to commonly observed complications such as cardiovascular diseases, individuals with T2DM exhibit an elevated susceptibility to the development of infections and tumors^5–10^. This indicates a potential impairment in the immune function of individuals with T2DM, possibly associated with hyperglycemia^11,12^. However, the precise mechanism of this impairment is yet to be fully elucidated.

Myeloid-derived suppressor cells (MDSCs) are a heterogeneous population of immature myeloid cells crucial for suppressing immune responses in various pathological settings^13^. MDSCs consist of two primary subpopulations: monocytic MDSCs (M-MDSCs) and granulocytic MDSCs (G-MDSCs). M-MDSCs exhibit a higher immunosuppressive capacity, and the current method for detecting M-MDSCs based on phenotype is well-established in humans^13,14^. Consequently, there is a substantial body of research exploring the relationship between M-MDSCs and various clinical diseases. Emerging data suggest that elevated levels of M-MDSCs in peripheral blood are often associated with conditions such as tumors, infections, and chronic inflammation^15–20^. Previous studies conducted by our team also demonstrate a significant association between elevated M-MDSCs and tumor progression, as well as a poor outcome in patients with acute myeloid leukemia and multiple myeloma^21,22^. These studies suggest that M-MDSCs may be closely related to the occurrence and development of tumor. Based on the above, we speculate that M-MDSCs may also associate with infection and tumor in T2DM patients, and conducted this study to detect the M-MDSCs level in T2DM patients and explore whether the M-MDSCs are correlated with the glycemia, and occurrence of infection and tumor.

## Results

### The frequency of M-MDSCs in healthy people

A total of 102 healthy individuals, aged between 40 and 65 years, underwent M-MDSC detection. There were no significant differences in age or sex distribution between the healthy individuals and patients with T2DM (*P* = 0.787 and 0.695 respectively, Table. 1). The median M-MDSCs frequency in healthy individuals was 0.93% (interquartile range, 0.54%–1.20%, Table. 2). We calculated the normal reference range and set the upper limit as a cut-off value (2.75%) according to the 95th quantile in healthy people. Patients with M-MDSC frequencies exceeding 2.75% were defined as having a high level of M-MDSCs.

**Table 1:** Clinical characteristics of T2DM patients and healthy control.

Characteristics^†^	All T2DM patients (n = 77)	GGC (n = 17)	PGC (n = 60)	Healthy Control (n = 102)	Normal value^‡^
FPG (mmol/L)	7.24 (5.76, 9.59)	5.26 (4.73, 5.88)	8.23* (6.35, 10.03)	5.17 (4.91, 5.37)	3.90–6.1
HbA1c (%)	8.70 (7.30, 10.30)	6.70 (6.40, 7.25)	9.55* (7.88, 10.88)	5.40 (5.20, 5.50)	4.0–6.0
Age (Years)	54.00 (48.00, 61.00)	49.00 (42.50, 58.00)	55.00* (50.25, 61.00)	53.00 (49, 57.5)	–
Gender, Male (%)	48.05% (n = 37)	47.06% (n = 8)	48.33% (n = 29)	46.07% (n = 47))	–
BMI	24.25 (22.64, 26.13)	24.13 (23.23, 25.24)	24.43 (22.50, 26.19)	19.65 (18.47, 20.31)	18.5–22.9
T2DM duration (Years)	9.50 (4.00, 10.75)	9.00 (1.50, 10.00)	10.00 (5.00, 12.00)	–	–
TG (mmol/L)	1.61 (1.06, 2.52)	1.51 (0.99, 2.03)	1.64 (1.19, 2.72)	1.24 (0.99, 1.61)	0.56–1.70
TC (mmol/L)	4.64 (3.75, 5.46)	4.35 (3.63, 5.13)	4.66 (3.84, 5.47)	4.90 (4.39, 5.69)	2.86–5.98
HDL-C (mmol/L)	0.99 (0.82, 1.18)	0.98 (0.80, 1.10)	1.00 (0.82, 1.21)	1.44 (1.22, 1.59)	1.04 ~ 1.55
LDL-C (mmol/L)	2.85 (2.35, 3.37)	2.78 (2.27, 3.22)	2.86 (2.33, 3.49)	2.59 (2.47, 3.05)	0 ~ 3.37
WBC(10^9^/L)	6.41 (5.76, 7.47)	6.32 (5.45, 8.57)	6.52 (5.86, 7.38)	5.67 (4.89, 7.10)	3.50–9.50
Lymphocyte (10^9^/L)	1.96 (1.56, 2.21)	2.14 (1.92, 2.51)	1.86 (1.49, 2.13)	1.93 (1.50, 2.50)	1.10–3.20
Neutrophil (10^9^/L)	3.81 (3.06, 4.93)	3.55 (3.06, 5.26)	3.90 (3.01, 4.92)	3.04 (2.46, 4.11)	1.80–6.30
Monocyte (10^9^/L)	0.45 (0.37, 0.57)	0.49 (0.35, 0.55)	0.44 (0.37, 0.59)	0.39 (0.30, 0.50)	0.1–0.60

^†^The Data are expressed as median (interquartile range).

^‡^The normal reference values were reported by the Clinical Examination Centre of the hospital.

*Compared with the GGC patient group (*P* < 0.05, assessed by non-parametric testing, Kolmogorov–Smirnov test).

*GGC* T2DM patients with good glycemic control (FPG ≤ 7.2 mmol/L and HbA1c ≤ 7.0%); *PGC* T2DM patients with poor glycemic control (FPG > 7.2 mmol/L or HbA1c > 7.0%).

**Table 2:** The M-MDSCs frequency in healthy people and T2DM patients.

Subjects	M-MDSCs frequency^†^ (%)
Healthy population (n = 102)	0.93% (0.54, 1.20)
All T2DM patients (n = 77)	2.32% (1.29, 4.21)*
GGC (n = 17)	1.65% (0.79, 3.76)
PGC (n = 60)	2.54% (1.39, 4.32)*

^†^The Data are expressed as median (interquartile range). *GGC* T2DM patients with good glycemic control (FPG ≤ 7.2 mmol/L and HbA1c ≤ 7.0%); *PGC* patients with poor glycemic control (FPG > 7.2 mmol/L or HbA1c > 7.0%). *Compared with the healthy population (*P* < 0.001). The frequency of M-MDSCs in all T2DM patients and the PGC group significantly higher than that in the healthy population (2.32% and 2.54% vs. 0.93%), but no significant difference between the GGC group and the healthy population (1.65% vs. 0.93%). The significance of the differences was assessed using non-parametric testing (Mann–Whitney *U* test).

### The frequency of M-MDSCs in T2DM patients

#### The clinical characteristics of T2DM patients

We enrolled 77 patients in the study. At the time of enrollment, the median duration since the first diagnosis of T2DM was 114 months (range, 1 month to 22 years), and none of the patients exhibited clinical signs of active infection or tumor. Other clinical characteristics, including TC, leukocyte counts, and their subsets were also analysed and compared. 77 patients were divided into good glycemic control (GGC) group and poor glycemic control (PGC) group (the details were provided in Methods section). The data revealed no significant difference between the GGC and PGC groups in T2DM duration, gender, body mass index (BMI), total cholesterol (TC), triglycerides (TG), low-density lipoprotein cholesterol (LDL-C), high-density lipoprotein cholesterol (HDL-C), except for fasting plasma glucose (FPG), haemoglobin A1c (HbA1c) and age (Table. 1).

### M-MDSCs in the PGC group significantly increased and correlated with glycemia level

**Figure 1:**
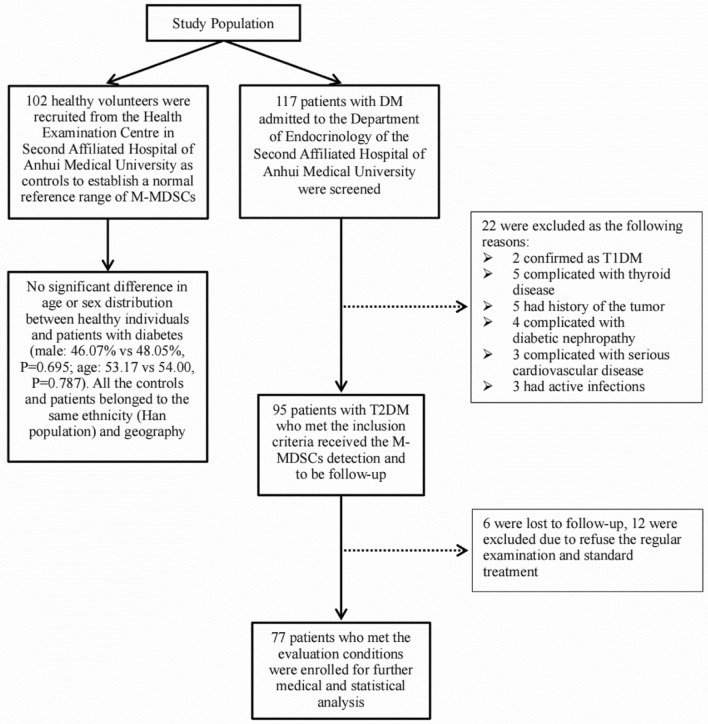
Flow chart of subjects screening. From May 2019 to June 2020, 102 healthy volunteers were recruited from the Health Examination Centre in Second Affiliated Hospital of Anhui Medical University as controls to establish a normal reference range of M-MDSCs, 107 patients with DM admitted to the Department of Endocrinology of the Second Affiliated Hospital of Anhui Medical University were screened, lastly 77 patients with T2DM who met the inclusion criteria and evaluation conditions were enrolled. All the controls and patients belonged to the same ethnicity (local Han population) and geography (residents of central Anhui province). There was no significant difference in age or sex distribution between healthy individuals and patients with diabetes (male: 46.07% vs. 48.05%, *P* = 0.695; age: 53.17 vs. 54.00, *P* = 0.787). T2DM diagnosis was based on the ADA standard (2018). All the patients must be received standard treatment and examination according to the guidelines for the prevention and treatment of diabetes in China (2017 edition).

**Figure 2:**
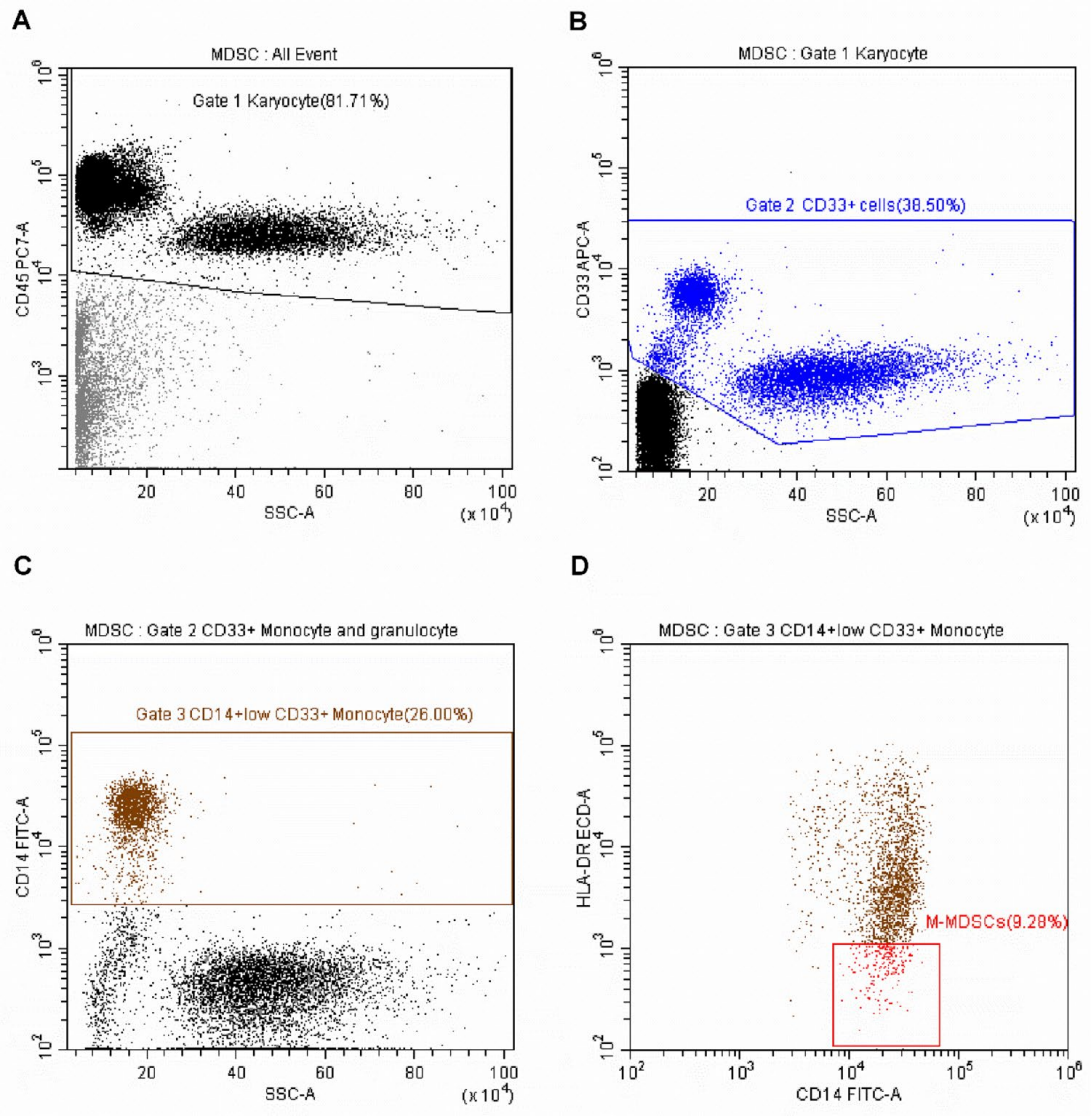
The detection of M-MDSCs in peripheral blood by flow cytometry. M-MDSCs was defined as CD14^+^CD33^+^CD45^+^HLA-DR^−^ Monocyte. The analysis method as follows: (**A**) All karyocytes (Gate 1) were selected by SSC and CD45 expression from all events. (**B**) The CD33^+^CD45^+^ cells (Gate 2) were selected from Gate 1. (**C**) The CD14^+^ Monocyte (Gate 3) were selected from Gate 2. (**D**) CD14^+^ HLA-DR^−^ Monocyte were selected from Gate 3 and the percentage of CD14^+^CD33^+^CD45^+^HLA-DR^−^ Monocyte represents the M-MDSCs level. All data were acquired and analyzed by Software CytExpert (2.0).

**Figure 3:**
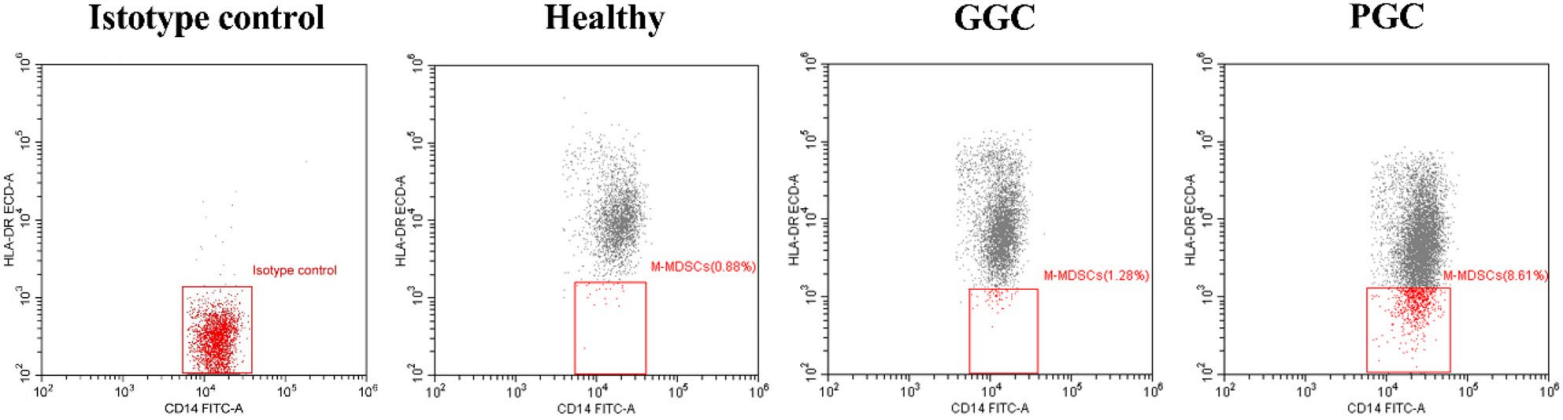
Demonstrating of M-MDSC frequencies in the healthy population and T2DM patients with good and poor glycemic control group. *The M-MDSCs frequencies were detected and analysed using FCM and CytExpert. The cells with a CD14^+^CD33^+^CD45^+^HLA-DR^−^ phenotype was defined as M-MDSCs (red box gate). Our results show that higher M-MDSCs frequency in the peripheral blood of T2DM patients, especially those with hyperglycemia, compared with healthy people.

**Figure 4:**
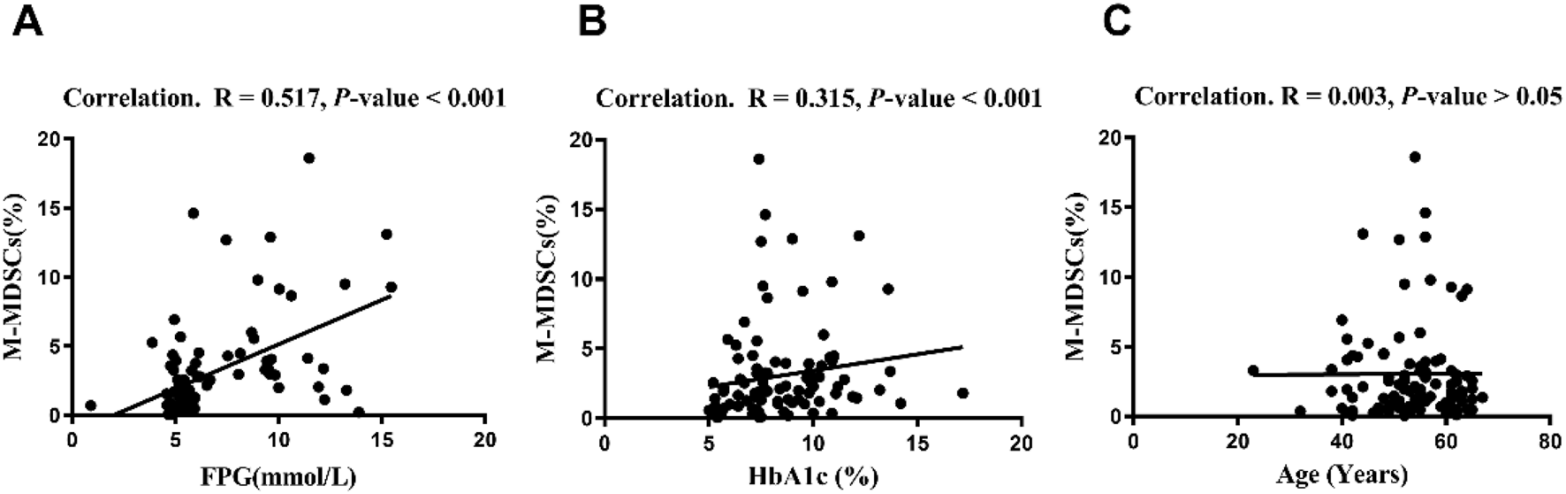
Correlations between M-MDSCs and FPG, HbA1c, and Age. *The correlations were analyzed using Spearman’s correlation. Our results show that there is a positive correlation between M-MDSCs and FPG (**A**), a positive correlation between M-MDSCs and HbA1c (**B**), and no correlation between M-MDSCs and Age (**C**) in T2DM patients.

The frequency of M-MDSCs in all T2DM patients was 2.32% (interquartile range, 1.29 to 4.21%), significantly higher than that in the healthy population (Figs. 1 and 2). However, when the patients were divided into GGC and PGC groups, the number of M-MDSCs in the PGC group was notably higher than in the healthy population (2.54%, 1.39–4.32% vs. 0.93%, 0.54–1.20%, *P* < 0.001), with no significant difference observed between the GGC group and the healthy population (1.65%, 0.79–3.76 vs. 0.93%, 0.54–1.20%) (Table. 2 and Fig. 3). To further understand the factors associated with M-MDSCs frequency in T2DM patients, we analysed the correlation between M-MDSCs and age, FPG, HbA1c, LDL-C, and BMI. We found a positive correlation between M-MDSCs and both FPG and HbA1c (*R* = 0.517 and 0.315, *P* < 0.001, respectively), but no correlation with the other indicators (Fig. 4).

### The elevated M-MDSCs in T2DM patients may pose a high risk of infection and tumor occurrence

During the follow-up period (median time: 7.6 months, range: 6–9.5 months), we found that the incidence of infection and tumor in T2DM patients with high M-MDSCs was 48.57% and 11.43% respectively, significantly higher than those in patients with normal M-MDSCs (19.05% and 0.00%, respectively) (Table. 3).

**Table 3:** The incidence of infection and tumor in T2DM patients with different M-MDSCs frequency during follow-up period.

Infection incidence (%)	Normal-MDSCs patients (N = 42)	High-MDSCs patients (N = 35)	*P *value
	19.05 (8/42)	48.57 (17/35)	0.006
Urinary tract infection	4	7	
Respiratory tract infection	4	4	
Ocular infection	0	2	
Oral infection	0	2	
Intra-abdominal infection	0	2	

Tumor incidence† (%)	0.00 (0/42)	11.43 (4/35)	0.039
Prostatic cancer	0	1	
Multiple myeloma	0	1	
Myelogenous leukemia	0	1	
Lung cancer	0	1	

Normal-MDSCs Patients, T2DM patients with normal M-MDSCs frequency (< 2.75%, N = 42); *High-MDSCs Patients* T2DM patients with high M-MDSCs frequency (≥ 2.75%, N = 35). The significance of the differences was assessed using the chi-square test or Fisher’s exact test.

^†^ Tumor incidence included patients who developed both infection and tumor during follow-up.

**Figure 5:**
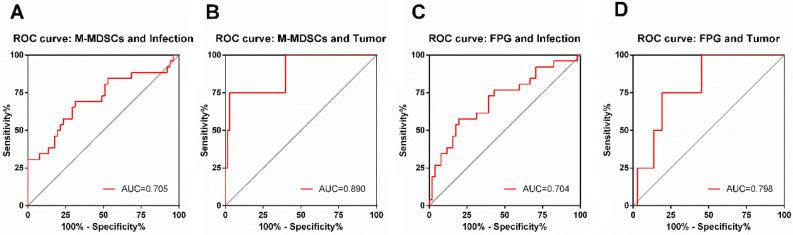
The ROC curves of M-MDSCs and FPG for predicting the occurrence of infection or tumor. *The predict value were analyzed by running the receiver operating characteristic (ROC). The area under the curve (AUC) and the best Youden index were calculated from the ROC curve. The results showed that M-MDSCs may be a significant biomarker for predicting the occurrence of infection and tumor, with optimal cut-off points of 2.80% and 11.24%, respectively, indicating their sensitivity and specificity (**A**,**B**). Again, FPG was significant for predicting infection and tumor (**C**,**D**). The optimal cut-off points of FPG for predicting infection and tumor was 8.91 mmol/L and 9.74 mmol/L, respectively. The data were analyzed in 77 T2DM patients.

To further explore the association between M-MDSCs and the occurrence of infection and tumor in T2DM patients, we analysed and compared M-MDSCs and other clinical indicators in T2DM patients with or without the occurrence of infection or tumor. In group A, which included patients with tumor (those who developed both infection and tumor during follow-up were also included in this group), the frequency of M-MDSCs was the highest and vastly higher than that in group B (patients with only infection) and group C (patients without any infection or tumor). The M-MDSCs levels in group B were higher than that in group C. As for other indicators, our analysis revealed significant differences in both FPG levels and lymphocyte counts. FPG levels were significantly higher in groups A and B compared to group C, with no significant difference observed between groups A and B. In contrast, group A exhibited significantly lower lymphocyte counts compared to groups B and C, while no significant difference between groups B and C (Table. 4). On this basis, we conducted a specific analysis to determine whether M-MDSCs, FPG, and lymphocyte counts could predict the development of infection or tumor in patients with T2DM using a receiver operating characteristic (ROC) and univariate logistic analysis. Regarding infection, M-MDSCs and FPG exhibited statistical significance (AUC = 0.705 and 0.704, respectively). For tumor, M-MDSCs and FPG also showed statistical significance (AUC = 0.89 and 0.798, respectively). There was no statistically significant difference in lymphocyte counts when predicting either infection or tumor. The best cut-off points for M-MDSCs to indicate the occurrence of infection and tumor were 2.80% and 11.24%, respectively. The best cut-off points for FPG to indicate infection and tumor occurrence were 8.91 mmol/L and 9.74 mmol/L, respectively (Fig. 5). Using these cut-off points and univariate logistic analysis, the relative risk (RR) for tumor occurrence in patients with M-MDSCs > 11.24% and FPG > 9.74 mmol/L was 43.20 (95% CI 5.432–343.63) and 10.58 (95% CI 1.175–95.384), respectively. The RR for infection occurrence in patients with M-MDSCs > 2.8% and FPG > 8.91 mmol/L was 2.50 (95% CI 1.235–5.060) and 2.84 (95% CI 1.534–5.244), respectively.

**Table 4:** Comparing of M-MDSCs, FPG and Lymphocyte count among patients with or without infection and tumor occurring.

Patient groups	M-MDSCs frequency (%)	FPG (mmol/L)	Lymphocyte count (10^9^/L)
Group A^†^ (n = 4)	12.89^a,b^ (5.35, 17.22)	10.66^b^ (8.05, 14.30)	0.63^a,b^ (0.37, 1.39)
Group B^‡^ (n = 22)	3.14^b^ (1.90, 6.58)	9.20^c^ (6.02, 11.58)	1.80 (1.17, 2.13)
Group C^§^ (n = 51)	1.95 (1.13, 3.37)	6.45 (5.49, 8.70)	1.97 (1.69, 2.44)

Data are expressed as median (interquartile range).

^†^Group A included patients who occurred tumor (patients who developed both infection and tumor during follow-up were also included in this group).

^‡^Group B included patients who only occurred infection.

^§^Group C included patients who developed neither infections nor tumor.

^a^Compared with group B, *P* < 0.05.

^b^Compared with group C, *P* < 0.05. Statistical differences were assessed using non-parametric tests.

## Discussion

This study aimed to explore the M-MDSCs level in T2DM patients and investigate their connections to glycemia, as well as the occurrence of infection and tumor. While MDSCs have been reported to be implicated in the incidence of diabetes, the focus has predominantly been on T1DM rather than T2DM^23–25^. Only a few studies suggest that the proportion of M-MDSCs in peripheral blood is higher in T2DM patients^26,27^, and there is almost no literature regarding the association of elevated MDSCs levels with poor glycemic control and the occurrence of infection and tumor. Our results suggest the frequency of M-MDSCs in peripheral blood of patients with T2DM was significantly higher than in the healthy population, particularly in PGC group compared with the GGC group, which were not mentioned in other study. Additionally, we observed a significant positive association between M-MDSCs and markers of glycemia, such as FPG and HbA1c levels. This observation suggests that hyperglycemia could be a cause of the elevated M-MDSCs levels in T2DM patients. Prior to this study, Li et al. co-cultured bone marrow cells from normal mice with IL-6, GM-CSF, and different concentrations of glucose to generate MDSCs in vitro, and observed that the induced MDSCs increased gradually with increasing glucose concentration. And mTOR signaling pathway plays a key role in the stimulation of MDSCs by high glucose^28^. These findings provide additional evidence supporting our study, suggesting that poor glycemic control and high blood glucose level may induce the proliferation and accumulation of M-MDSCs.

Furthermore, we were surprised to find that the occurrence of infection and tumor in T2DM patients was associated with abnormally increased M-MDSCs. Patients with tumors exhibited the highest M-MDSCs frequency, followed by patients with only infections and those without any infection or tumor, these results imply that higher M-MDSCs levels in T2DM patients might indicate an increased risk of developing infections and tumors. It may due to the formation of immunosuppressive microenvironments and suppressing T cells induced by M-MDSCs accumulation which has been supported by many researches^29–35^. Wang et al.^36^ reported that MDSCs from T2DM mice inhibited the proliferation and modulated the cytokine secretion of autologous CD4^+^ T cells in vitro. This aligns with the known function of MDSCs in other pathological states, such as tumor. M-MDSCs can inhibit T cell proliferation and activation by producing nitric oxide and reactive oxygen species (ROS), considered a core feature of M-MDSCs^34^. Therefore, we speculate that the prolonged presence of M-MDSCs may impair immune function, thereby creating conditions for tumorigenesis and infection in patients with T2DM.

We also examined other clinical indicators and found that FPG levels and lymphocyte counts were significantly dysregulated in patients with tumors and infections compared to those without any infection or tumor. Through ROC analysis and univariate logistic regression, M-MDSCs and FPG showed statistically significant predictive value for both infection and tumor occurrence. Several studies have suggested that blood glucose and M-MDSCs could serve as independent predictors of different disease states, demonstrating their potential as biomarkers^21,22,33–38^. In this study, especially M-MDSCs, may possess the capability as a stronger predictor for tumorigenesis and infectious in T2DM patients.

Previous studies have demonstrated that chronic low-grade inflammation induced by adipose tissue expansion and obesity leads to insulin resistance, impaired insulin secretion, and ultimately, hyperglycemia^4,39,40^. Simultaneously, inflammation can also activate MDSCs and drive their accumulation and suppressive activity in order to curb excessive, and potentially harmful, immune responses^30,39^. Therefore, the MDSCs may protect the pancreatic islets from inflammation injure during the early stages of diabetes^38^. However, with the progression of T2DM, M-MDSCs may play a dual role. The abnormally increased M-MDSCs under the poor glycemic control and hyperglycemia condition may cause T2DM patients become more sensitive to infection and tumor. Our results shed new light on the pathogenesis of T2DM, help to understand why T2DM patients are susceptible to infection and tumor and providing novel insights for future prevention and treatment of T2DM^3,28,38,41^.

In conclusion, our study reports for the first time that M-MDSCs significantly increase, mainly in T2DM patients with poor glycemic control, and there is a positive correlation between M-MDSCs and glycemic level. T2DM patients with abnormal increased experience a higher occurrence of infection and tumor. These findings provide valuable information for further exploring the immune mechanisms of T2DM, and revealed the importance of glycemic control. Moreover, the molecular mechanism of the relationship between the M-MDSCs and hyperglycemic needs to be elucidated in the future, and more clinical evidence is required to further prove the significance and feasibility of M-MDSCs predicting risk of infection and tumor in T2DM patients.

## Methods

This research is a hospital-based case–control study. A prospective observational pilot study was also conducted. The study protocol was approved by the institutional review board and the Ethics Committee of the Anhui Medical University (approval number: YJ-YX2020-004). Written informed consent was obtained from all patients and volunteers in accordance with the Declaration of Helsinki.

### Study population

After obtaining informed consent, from May 2019 to June 2020, 102 healthy volunteers were recruited from the Health Examination Centre in Second Affiliated Hospital of Anhui Medical University as controls to establish a normal reference range of M-MDSCs in our laboratory. 117 diabetes patients admitted to the Department of Endocrinology of the Second Affiliated Hospital of Anhui Medical University were screened, finally 77 eligible cases were included in this study (Fig. 1). All the controls and patients belonged to the same ethnicity (local Han population) and geography (residents of central Anhui province). The diagnosis of T2DM was based on The American Diabetes Association (ADA) Standards (2018)^42^, and every T2DM patient received standard treatment according to the guidelines for the prevention and treatment of diabetes in China (2017 edition). Patients with the following conditions were excluded: type 1 diabetes mellitus (T1DM), active infection, history or the possibility of other autoimmune diseases, tumor, immunologic deficiency diseases, and other serious complications.

### Flow cytometric analysis for M-MDSCs detection

2–5 mL peripheral blood was collected from healthy volunteers and patients. The samples were anti-coagulated with EDTA and used to detect M-MDSCs frequency within 4 h. 100 μL peripheral blood was mixed with Fluorescein isothiocyanate (FITC)-conjugated CD14-specific monoclonal antibodies (mAb), Phosphatidylethanolamine (PE)-conjugated HLA-DR mAb, DX-5-allophycocyanin (APC)-conjugated CD33 mAb, and phycoerythrin-Cy7 (PC7)-conjugated CD45 mAb or with their appropriate isotype controls and incubated in the dark for 15 min at room temperature. Next, the red blood cells were lysed using ammonium chloride solution (8.3 mg mL-1, volume ratio of 1–9), and samples were detected immediately by flow cytometer FC-500 and analysed using CXP 2.0 software (Cytomics FC-500, CXP 2.0 software, Beckman Coulter Ltd, California, USA).

The above-mentioned mAbs specific for human surface antigens were purchased from Beckman Coulter Immunotech (Beckman Coulter Immunotech, Marseille, France): FITC-labelled CD14 (clone 116), PE-labelled HLA-DR (clone B8.12.2), APC-labelled anti-CD33 (clone 13B8.2), PC7-labelled CD45 (clone J.33), and their appropriate isotype controls.

Cells with CD14^+^CD33^+^CD45^+^HLA-DR^−^ phenotype were defined as M-MDSCs^13,14^, which percentage in all monocytes (referred to as “frequency”) represents the M-MDSCs level. The specific detection and analysis methods were shown in Fig. 2A–D.

### Clinical chemistry and microscopic parameters

Other routine metabolic markers, such as FPG, HbA1c, TC, TG, LDL-C, HDL-C, and complete blood counts etc., were all measured and reported by the Clinical Examination Centre of the hospital. Blood cells were counted by an automatic hematology analyzer (SysmeXE-2100, Sysmex Corporation, Kobe, Japan). Plasma biochemical data were assessed by an automatic biochemistry analyzer (AU-5800, Beckman Coulter Ltd, California, USA).

### Clinical data collection and follow-up of patients

Clinical data of patients with M-MDSCs were collected, such as age, BMI, T2DM duration, disease history, and other related examination results. All enrolled patients were divided into two various glycemic control groups according to their glucose levels. According to the ADA standards (2018)^43^, patients with FPG > 7.2 mmol/L or HbAlc > 7.0% were defined as the PGC group, while patients with FPG ≤ 7.2 mmol/L and HbAlc ≤ 7.0% were assigned to the GGC group. A prospective observational pilot study was performed, all the T2DM patients were followed up at least 6 months after M-MDSCs detection. The selection of the follow-up time was mainly based on similar clinical studies and the related suggestion^44,45^. During the follow-up period, the diagnosis of patients with infection or tumor occurrence was based on clinical manifestations, elevated inflammatory factors or tumor serological indicators, imaging, and pathological examination, and confirmed by a clinical specialist.

### Statistical analysis

The sample size of this research was calculated using sample size formula for hospital-based case–control study. To compared the difference of M-MDSCs frequency among the T2DM patients and healthy population, the Two-sample unequal variance T-test analysis was performed by PASS 2021 (PASS software, Kaysville, Utah, USA). The sample formula was listed below.

$\mathrm{The sample formula}, t=\frac{\overline{X1}-\overline{X2}}{Sp\sqrt{\frac{1}{N1}+\frac{1}{N2}}}$. $\overline{X1} and \overline{X2}$ represent the mean of M-MDSCs frequency in T2DM patients and healthy controls, respectively. The N_1_ and N_2_ represent the sample size of T2DM patients and healthy controls. The Sp represent the pooled standard deviation. The means of M-MDSCs frequency were derived from the existing literature and our previous works^22,27,36^. We assumed the M-MDSCs frequency in T2DM patients was 1.5% ± 1.0% and 1.0% ± 0.2% in healthy population. With α = 0.05 and power = 90%, there was 46 individuals in each group. Therefore, after considering possible dropout rates (20%), a minimum number of 58 individuals in T2DM and healthy controls groups were required.

All statistical analyses were performed using the SPSS version 22.0 software (SPSS, Inc., Chicago, USA). Quantitative data were presented as medians and quartiles [M (Q_25_, Q_75_)]. Kolmogorov–Smirnov test was performed for normality test. Differences among groups were analysed using one-way or two-way ANOVA, followed by Student–Newman–Keuls (SNK) multiple range test or Tukey's multiple comparison test if the data followed a normal distribution. The data that did not comply with the above-mentioned conditions were analysed using the Mann–Whitney U test or Kruskal-Walli’s test. Correlations were analysed using Spearman’s test. ROC curves were plotted for predictive analysis and cut-off values. Univariate logistic analysis was used to explore risk factors for the occurrence of infection or tumor. Chi-square and Fisher’s exact tests were used to compare the incidence and RR between groups. Statistical significance was set at* P* < 0.05.

## Data Availability

Data is available from the corresponding author on reasonable request.
